# Whole-body dual-energy X-ray absorptiometry demonstrates better reliability than segmental body composition analysis in college-aged students

**DOI:** 10.1371/journal.pone.0215599

**Published:** 2019-04-22

**Authors:** Petr Kutáč, Václav Bunc, Martin Sigmund

**Affiliations:** 1 Human Motion Diagnostics Center, University of Ostrava, Ostrava, Czech Republic; 2 Faculty of Physical Education and Sport, Charles University, Praha, Czech Republic; 3 Application Centre BALUO, Faculty of Physical Culture, Palacký University, Olomouc, Czech Republic; Kennesaw State University, UNITED STATES

## Abstract

Dual-energy X-ray absorptiometry (DXA) is rapidly becoming more accessible and popular as a technique to monitor body composition. The reliability of DXA has been examined extensively using a number of different methodological approaches. This study sets up to investigate the accuracy of measuring the parameters of body composition (BC) by means of the whole-body and the segmental DXA method analysis with the typical error of measurement (TEM) that allows for expressing the error in the units of measure. The research was implemented in a group of 63 participants, all of whom were university students. Thirty-eight males (22.6±2.9 years, average body mass 77.5±8.4 kg) and 25 females (21.4±2.0 years, average body mass 58.6±7.2 kg) were recruited. The measured parameters included body mass (BM), fat-free mass (FFM), body fat (BF), bone mineral content (BMC), bone mineral density (BMD). For the whole-body analysis, the determined TEM was: BM at the level of 0.12 kg in females and 0.29 kg in males; BF 0.25kg and 0.44% females, 0.52 kg and 0.66% males; FFM 0.24 kg females and 0.42 kg males; BMC 0.02 kg females and males; BMD 0.01g/cm^2^ females and males. The TEM values in the segmental analysis were: BF within the range of 0.04–0.28 kg and 0.68–1.20% in females, 0.10–0.36 kg and 0.72–1.94% in males; FFM 0.08–0.41 kg females and 0.17–0.86 males, BMC 0.00–0.02 kg females and 0.01–0.02 kg males in relation to the body segment (upper limb, trunk, lower limb). The BMD value was at the level of 0.01–0.02g/cm^2^. The study results showed high reliability in measuring body composition parameters using the DXA method. The whole-body analysis showed a higher accuracy of measurement than the segmental. Only the changes that are greater than the TEM, or the upper bound (95%) of the confidence interval of the measurement can be considered demonstrable when interpreting repeated measurements.

## Introduction

The analysis of body composition has become common in the assessment of an organism’s condition. The values of body composition parameters are used to assess the health of an individual, the quality of nutrition and overall fitness. These values are also used in sports to assess the effects of training and nutrition on the changes in the individual components of the athlete’s body weight, or the changes in the individual components during the competition season [1–6]. To analyze body composition, indirect methods are used because a direct method would be difficult to execute in a living person. Currently, direct measurement of body segment inertial parameters (BSIPs) on living humans is possible using medical imaging technologies such as gamma-ray scanning [7,8], computed tomography imaging (CT) [9,10] and magnetic resonance imaging (MRI) [11–13]. Although accurate, they are not widely used in biomechanics due to costs, labor demands during data processing, limited accessibility and/or exposure of subjects to high doses of radiation. The indirect methods include field techniques and referential methods. The results of the analysis depend on the method and equipment used as well as the current condition of the individual [14–17]; in addition, the results obtained by the same method but different devices differ from one another [18–21]. The prediction equations for determining the measured parameters are the basic problem, each manufacturer uses their own equations in the device software. The results are influenced by the measurement errors. With regard to their effect, errors can be divided into systematic and random [22]. A researcher cannot control random errors and systematic errors distort the result in the same way provided that the same conditions of measurement are observed. In addition to the inter- and intra-examiner errors, there are also errors related to the methodology, device and measuring instruments. Thus, to correctly interpret the results of the measurement (especially in repeated measurements), the knowledge of the errors in the chosen method and device is necessary. In the biomedical sciences field, it is recommended to express the error using the typical error of measurement (TEM) [23]; unlike the commonly used reliability coefficient, TEM allows for expressing the existing error directly in the units used in the experiment. To calculate TEM, Hopkins [23] recommends performing three repeated (consecutive) measurements.

Dual-Energy X-Ray Absorptiometry (DXA) is commonly used for body composition measurement and in the assessment of athletes. DXA is a recent medical-imaging technique with potential for direct measurement of BSIPs in living subjects. It is similar to gamma-ray scanning as it relies on the attenuation of radiation beams passing through the body to measure surface density. The main difference is that DXA uses two X-ray intensities which allow measurement of bone mineral and soft tissue masses separately (the latter includes fat and lean tissue masses) [24,25]. Hence, it is used primarily to determine bone mineral density and body composition in vivo [24–28]. Recently, it has been used to estimate segment mass, center of mass position in the frontal plane, and moment of inertia about the center of mass [29–31]. DXA is accurate, noninvasive, low cost, low radiation emitting method that is faster to analyze than gamma-ray scanning and other imaging methods. DXA provides information on three compartments of body composition, according to the terminology “fat mass,” “lean mass” or the “fat-free soft tissue” and “bone mineral content.” A series of studies have confirmed DXA as being the benchmark of body composition measurements, the results obtained by this method are used as a criterion for detecting the external criteria validity of the field methods [14–16,32,33]. However, measurement errors have to be considered even with this method. To calibrate the devices and to verify the method, a phantom supplied by the manufacturer can be used (e.g. spine phantom of vertebrae L_1_-L_4_). Still, the result of the measurement can be influenced by the person who is measured. A human is a biological subject for whom individual variability is typical. Therefore, the mere use of the phantom to determine the accuracy of measurement is not enough. There are many studies that deal with the issue of DXA measurements. Some of them compare results measured by the various systems [18,20,34,35], others deal with the validity of the DXA method when compared to MRI [36], or the effect of the participant’s body size on the accuracy of the measurement [37]. There are also studies that look at the reproducibility of the DXA method. These studies assess the effect of the diet on changes in body composition [38], the reliability of the method using the intraclass correlation coefficient (ICC) with a two-week interval [39] or using ICC and the standard error measurement percentage on a limited number of people [40]. In order to interpret the results of the repeated measures or assess the uniformity of the segmental distribution of body composition components, the knowledge of the magnitude of measurement-related errors in commonly used units is required. This allows for a proper assessment of the differences found in the results. The above mentioned studies did not assess the errors of measurement described.

Therefore the purpose of this study was to verify the reliability and to determine error of measurement in whole-body and segmental analysis of body composition when using the DXA method.

## Materials and methods

### Participants

Sixty-three participants were recruited, 38 males (22.6±2.9 years) with an average body height of 180.8±6.3 cm, body mass 77.5±8.4 kg and BMI 23.7±2.3 kg/m^2^, and 25 females (21.4±2.0 years) with an average body height of 168.3±6.7 cm, body mass 58.6±7.2 kg and BMI 20.7±1.8 kg/m^2^. All study participants were healthy, they did not take any medicine or food supplements. Participants were graduate students enrolled in the teacher preparation program for physical education. They participated voluntarily in the study and were informed about the procedure of the study in advance. All participants were required to undergo a thorough medical check annually by a registered physician. All participants also signed the informed consent of participation in the study. The study was approved by the ethics committee of the University of Ostrava (number 019/0000798) and it was in compliance with the Helsinki Declaration.

### Procedures

**Fig 1:**
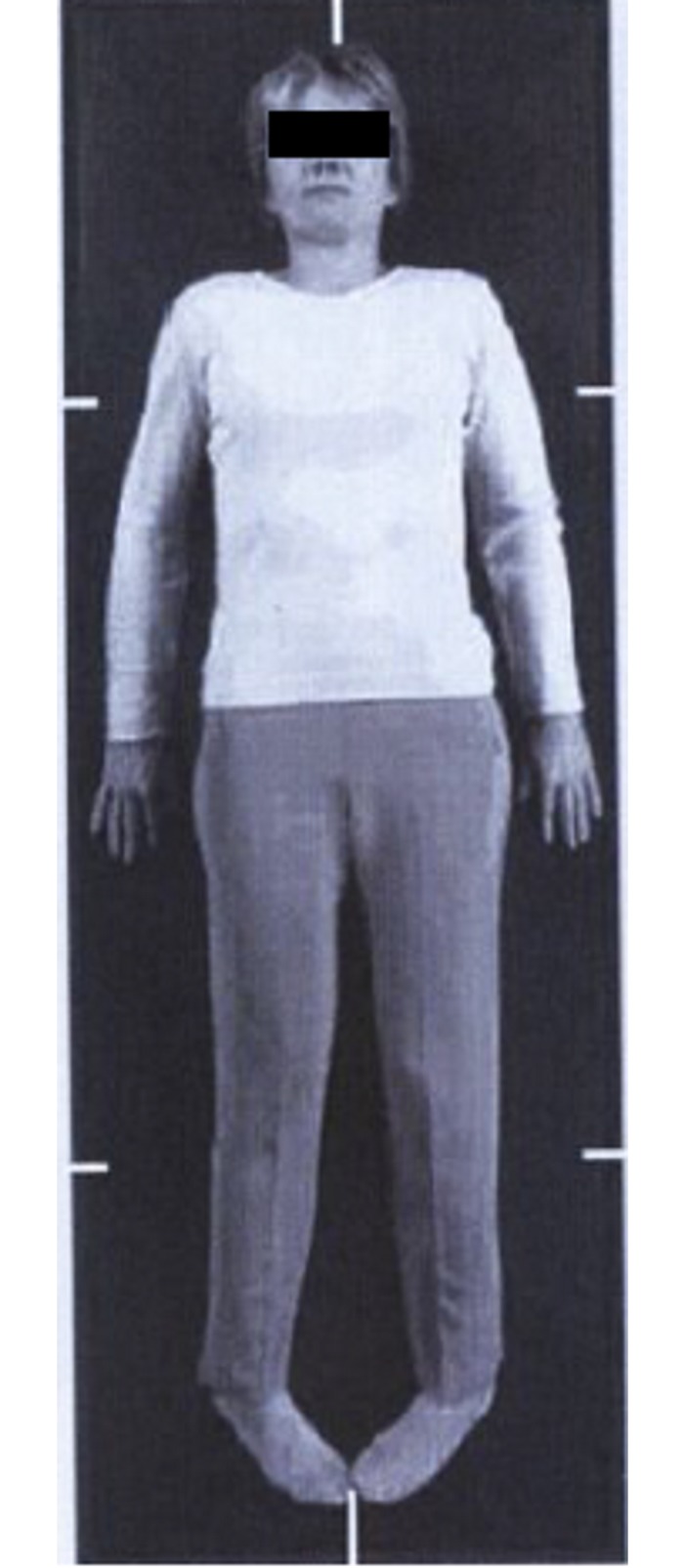
Position of the participant during measurement [42].

The measurements of the basic anthropometric parameters of body height (BH) and body mass (BM), which are the input parameters for measuring body composition using the DXA system, were taken using a stadiometer with a digital scale, InBody BSM 370 (Biospace, South Korea). The manufacturer states the accuracy of measurement at 0.1 cm for BH and 0.1 kg for BM. Whole body scans were performed using DXA (Hologic Discovery A, Waltham, MA) in order to quantify the magnitude and quality of full-body mass distribution (lean, fat, bone and total). Participants assumed a stationary, supine position on the scanning bed with both arms pronated by their side. The position of the participant during the measurement is shown in Fig 1. To ensure consistent and reproducible positioning, the DXA operator manually assisted participants in order to: 1) straighten the head, neck and torso parallel to the long axis of the scan bed; 2) position the shoulders and pelvis perpendicular to the long axis of the scan bed; 3) place both arms in pronation by their side; 4) place legs at shoulder width with 45° internal rotation; and 5) fixate feet together using strapping tape to minimise incidental movement and for the participants comfort within the DXA scanning zone. This has been shown to produce a scan-rescan coefficient of variation under 1% in our laboratory for body composition components [41]. All measurements were done in the morning (8.00–10.00 a.m.), the participants were not involved in any medium and high-intensity physical activity one day prior to the measurement and they were all recommended to follow a regular intake of liquids.

**Fig 2:**
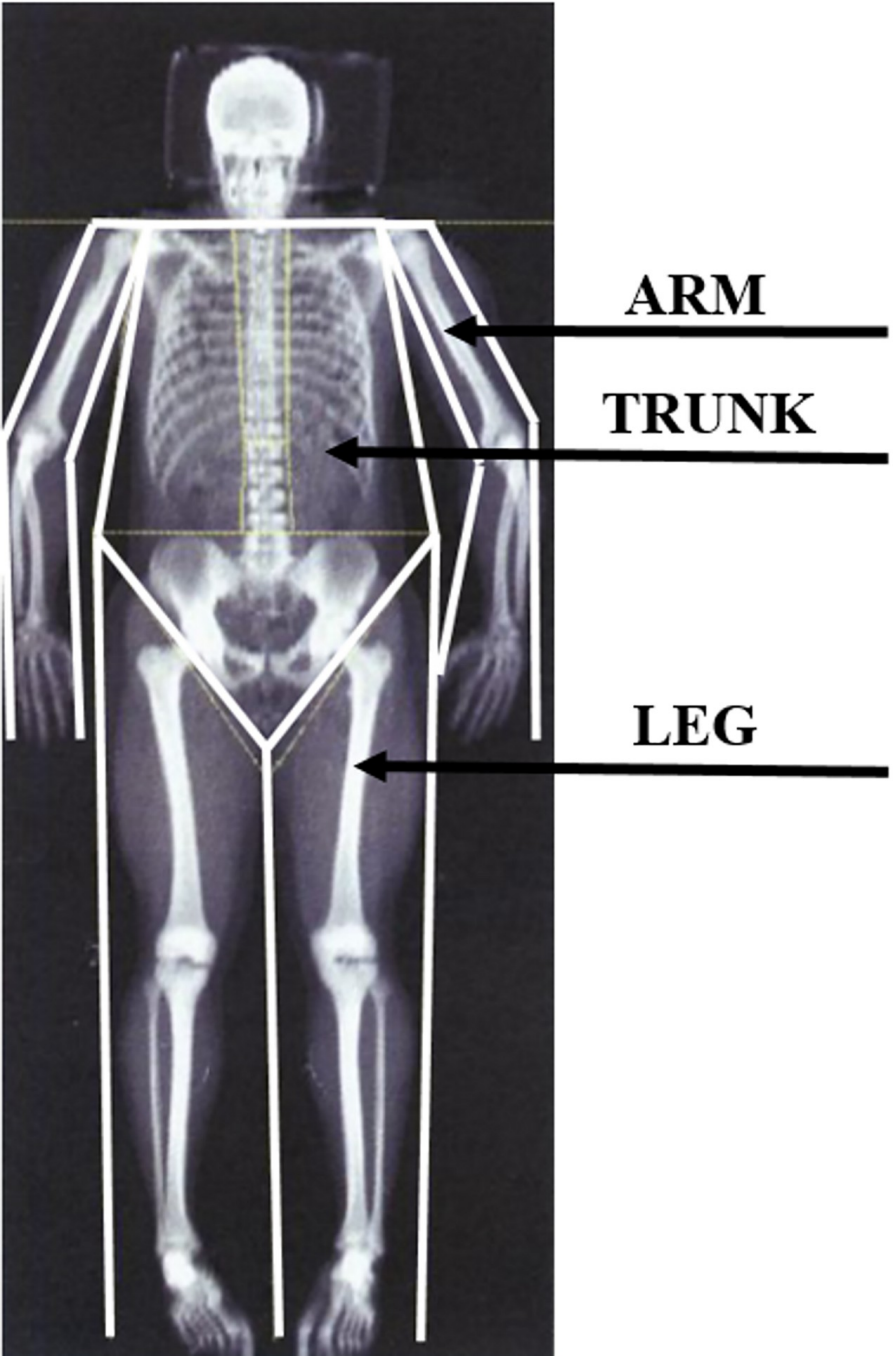
Body scan [42].

Upon scan completion, a two-dimensional image was automatically generated for scan analysis purposes. Using the in-built scan analysis software (Version 12.4; QDR for Windows, Hologic, Waltham, MA), the full-body images were separated into axial and appendicular regions using the predefined and mandatory whole body model as required by the software [42]. Further analysis was subsequently performed to manually identify and assess appendicular segmental masses. Specifically, using the sub-region analysis tool, customised regions-of-interest (ROI) were drawn to capture twelve segments: the left upper arm, right upper arm, left forearm, right forearm, left hand, right hand, left thigh, right thigh, left shank, right shank, left foot and right foot regions. Each participant was measured three times consecutively, according to the recommendations by Hopkins [23]. The third measurement verifies the second one, which guarantees stable conditions. The interval between the repeated measurements was 3 minutes at the most. After each measurement, the participant sat up on the measuring table, the laboratory technician placed the participant in the corresponding position for measuring again and commenced measuring. This procedure was used to verify not only the reproducibility of the device measurement but also the accuracy of the laboratory technician when positioning the participants. The parameters measured were body fat (BF), fat-free mass (FFM), bone mineral content (BMC), bone mineral density (BMD) and their segmental distribution of the arms, legs, and the trunk. The body scan is presented in Fig 2.

Subject’s hydration may significantly affect its DXA estimates and thus has to be thoroughly checked at least 24 hours prior to the measurement to ensure relevant interpretation of the DXA results both in the whole-body and segmental analysis.

### Statistical analysis

The accuracy of the DXA system was assessed using the TEM [23]. The TEM for trial pairs was calculated from the selective standard deviation in differences between two trials of the participant and divided by the root of the number of trials. The resulting value of the typical error is calculated from the root of the dot products of the squares of typical errors (TE^2^) of trial pairs and the degrees of freedom (male: Df = 37, female: Df = 24), divided by the total sum of the degrees of freedom [23].

$$\text{Resulting}\;\text{typical}\;\text{error}\;\text{of}\;\text{measurement:}\;\frac{\sqrt{\sum_{i}TE_{i}^{2}\cdot Df_{i}}}{\sum_{i}Df_{{}_{i}}}$$

With regard to the fact that these were repeated measurements of the same people, intraclass correlation (ICC) [43] was used to assess the correlation of the resulting values of the monitored parameters between the repeated measurements.

To assess the practical significance of the found differences in the mean values between the individual measurements, the effect size according to Cohen was used (Cohen’s *d)*. The *d* value at the level of 0.2 indicates a minor change, 0.5 an intermediate change and 0.8 a major change [22]. The calculation was based on the values of the measured body composition parameters from three consecutive measurements of each participant. Statistical analysis was performed using a statistical spreadsheet [44]. To assess the statistical significance of the differences in the mean TEM values for males and females in the monitored parameters, the parametric two-selection t-test was used. The level of statistical significance was selected at α = 0.05. The statistical processing of the results was performed using IBM SPSS Statistics (Version 21 for Windows; IBM, Armonk, NY, USA).

## Results

The results present the mean input values of the whole-body and segmental analyses (the segmental analysis did not include the head) and the resulting values of the TEM. The mean input values were acquired in three repeated measurements of the individual participants, as described in the measurement procedure. The reproducibility of the measurement results using DXA was verified using TEM. The values of the whole-body analysis are presented in Table 1, the segmental analysis in Table 2. The results of the DXA measurement outcome reproducibility are presented in Tables 3 and 4. The TEM and ICC values, which are presented in Tables 3 and 4, are calculated as the mean values of two consecutive comparative measurements (Trial 1–2, 2–3).

**Table 1:** Input values of repeated measurements presented by the mean and standard deviation—Whole body analysis.

Parameters	Trial 1	Trial 2	Trial 3
	M±SD	M±SD	M±SD
	**Male**		
**BM**_**DXA**_ **(kg)**	76.99±8.29	76.91±8.10	76.92±8.23
**BF (kg)**	13.54±3.50	13.48±3.44	13.47±3.45
**BF (%)**	17.57±3.91	17.51±3.81	17.49±3.78
**FFM (kg)**	60.39±7.30	60.37±7.11	60.38±7.14
**BMC (kg)**	3.05±0.30	3.06±0.31	3.06±0.31
**BMD (g/cm**^**2**^**)**	1.29±0.08	1.29±0.08	1.29±0.08
	**Female**		
**BM**_**DXA**_ **(kg)**	58.37±6.96	58.39±7.04	58.46±7.03
**BF (kg)**	15.30±3.07	15.31±3.17	15.31±3.17
**BF (%)**	26.20±4.17	26.20±4.32	26.16±4.34
**FFM (kg)**	40.76±5.49	40.77±5.54	40.84±5.53
**BMC (kg)**	2.31±0.33	2.31±0.33	2.31±0.33
**BMD (g/cm**^**2**^**)**	1.16±0.09	1.15±0.09	1.15±0.09

The mean values of BM_DXA_ shown in Table 1 were acquired by calculating the sum of the BMC, FFM and BF, and not by weighing.

**Table 2:** Input values of repeated measurements presented by the mean and standard deviation—Segmental analysis.

Segments	Parameters	Male			Female		
		Trial 1	Trial 2	Trial 3	Trial 1	Trial 2	Trial 3
		M±SD	M±SD	M±SD	M±SD	M±SD	M±SD
**LARM**	**BF (kg)**	0.68±0.21	0.70±0.21	0.67±0.20	0.75±0.18	0.74±0.20	0.75±0.21
	**BF (%)**	15.42±4.16	15.77±4.37	15.99±4.00	27.19±5.66	27.03±5.84	27.00±6.04
	**FFM (kg)**	3.54±0.63	3.54±0.59	3.58±0.62	1.87±0.31	1.86±0.32	1.89±0.34
	**BMC (kg)**	0.20±0.03	0.20±0.03	0.21±0.03	0.13±0.02	0.13±0.02	0.13±0.02
	**BMD (g/cm**^**2**^**)**	0.89±0.06	0.89±0.06	0.88±0.05	0.75±0.05	0.74±0.05	0.75±0.05
**RARM**	**BF (kg)**	0.69±0.22	0.68±0.21	0.69±0.22	0.74±0.19	0.73±0.21	0.73±0.20
	**BF (%)**	14.99±3.89	15.14±3.86	15.20±3.79	25.68±5.99	25.51±6.48	25.34±6.15
	**FFM (kg)**	3.66±0.54	3.59±0.48	3.61±0.55	2.00±0.37	2.00±0.38	2.02±0.40
	**BMC (kg)**	0.21±0.03	0.20±0.03	0.20±0.03	0.15±0.02	0.15±0.02	0.15±0.02
	**BMD (g/cm**^**2**^**)**	0.91±0.07	0.93±0.08	0.94±0.08	0.77±0.05	0.77±0.06	0.77±0.06
**TRUNK**	**BF (kg)**	6.30±1.79	6.29±1.90	6.12±1.68	6.25±1.67	6.29±1.67	5.76±1.60
	**BF (%)**	17.52±4.24	17.43±4.31	17.42±4.16	23.00±4.97	23.10±5.09	22.50±5.14
	**FFM (kg)**	28.76±3.70	28.82±3.76	28.18±3.59	20.15±2.54	20.18±2.56	19.10±2.5
	**BMC (kg)**	0.88±0.12	0.88±0.12	0.87±0.11	0.66±0.10	0.66±0.09	0.63±0.10
	**BMD (g/cm**^**2**^**)**	1.07±0.09	1.06±0.09	1.06±0.09	0.97±0.07	0.96±0.07	0.96±0.07
**LLEG**	**BF (kg)**	2.26±0.67	2.22±0.61	2.28±0.71	3.23±0.65	3.22±0.69	3.45±0.72
	**BF (%)**	17.16±4.50	16.91±4.18	16.84±4.42	30.99±4.35	30.90±4.48	30.92±4.51
	**FFM (kg)**	10.25±1.28	10.27±1.26	10.57±1.47	6.76±1.10	6.76±1.10	7.26±1.13
	**BMC (kg)**	0.62±0.07	0.63±0.08	0.63±0.08	0.43±0.06	0.43±0.06	0.45±0.07
	**BMD (g/cm**^**2**^**)**	1.49±0.12	1.49±0.12	1.48±0.13	1.23±0.10	1.23±0.10	1.23±0.10
**RLEG**	**BF (kg)**	2.34±0.72	2.32±0.68	2.44±0.76	3.28±0.68	3.27±0.72	3.55±0.78
	**BF (%)**	17.63±4.81	17.55±4.64	17.82±4.70	31.29±4.54	31.20±4.72	31.26±4.93
	**FFM (kg)**	10.27±1.36	10.26±1.31	10.54±1.52	6.77±1.23	6.76±1.23	7.34±1.30
	**BMC (kg)**	0.64±0.08	0.64±0.08	0.6±0.09	0.45±0.08	0.45±0.08	0.46±0.08
	**BMD (g/cm**^**2**^**)**	1.52±0.12	1.51±0.14	1.51±0.04	1.26±0.11	1.26±0.11	1.25±0.12

The differences between male and female subjects in the absolute values of anthropometric parameters were significant; the differences in the relative values of BMD expressed in g/cm^2^ were not significant.

**Table 3:** The mean typical error of measurement value, lower and upper confidence limit and intraclass correlation—Whole body analysis.

Parameters	TEM (95% CI)		ICC	
	Male	Female	Male	Female
**BM**_**DXA**_ **(kg)**	0.29 (0.25, 0.34)	0.12** (0.10, 0.15)	0.99	1.00
**BF (kg)**	0.52 (0.45, 0.62)	0.25** (0.21, 0.32)	0.98	0.99
**BF (%)**	0.66 (0.57, 0.79)	0.44** (0.37, 0.55)	0.98	0.99
**FFM (kg)**	0.42 (0.36, 0.50)	0.24* (0.20, 0.30)	0.99	0.99
**BMC (kg)**	0.02 (0.02, 0.03)	0.02 (0.02, 0.02)	0.99	0.99
**BMD (g/cm**^**2**^**)**	0.01 (0.01, 0.01)	0.01 (0.01, 0.01)	0.99	0.99

*p<0.05;

**p<0.01

**Table 4:** The mean typical error of measurement value, lower and upper confidence limit and intraclass correlation—Segmental analysis.

Parameters	Statistical characteristics	RARM		RLEG		TRUNK	
		Male	Female	Male	Female	Male	Female
**BF (kg)**	TEM (95% CI)	0.10 (0.09, 0.12)	0.04** (0.04, 0.05)	0.22 (0.19,0.26)	0.12** (0.10, 0.14)	0.36 (0.31, 0.43)	0.28 (0.23, 0.35)
	ICC	0.82	0.95	0.89	0.98	0.96	0.97
**BF (%)**	TEM (95% CI)	1.94 (1.67, 2.31)	1.20** (1.00, 1.49)	1.48 (1.28,1.77)	0.68** (0.56, 0.84)	0.72 (0.62, 0.86)	0.71 (0.59, 0.89)
	ICC	0.85	0.97	0.90	0.98	0.98	0.98
**FFM (kg)**	TEM (95% CI)	0.17 (0.14, 0.20)	0.08** (0.07, 0.10)	0.49 (0.42, 0.59)	0.23** (0.19, 0.28)	0.86 (0.74, 1.03)	0.41** (0.34, 0.51)
	ICC	0.88	0.96	0.87	0.96	0.97	0.98
**BMC (kg)**	TEM (95% CI)	0.01 (0.01, 0.01)	0.00 (0.00, 0.00)	0.02 (0.02, 0.02)	0.01 (0.01, 0.01)	0.02 (0.02, 0.02)	0.02 (0.01, 0.02)
	ICC	0.96	0.98	0.96	0.98	0.98	0.97
**BMD (g/cm**^**2**^**)**	TEM (95% CI)	0.02 (0.02, 0.03)	0.01 (0.01, 0.01)	0.02 (0.02, 0.02)	0.01 (0.01, 0.01)	0.02 (0.01, 0.02)	0.02 (0.01, 0.02)
	ICC	0.92	0.97	0.98	0.99	0.98	0.95

*p<0.05;

**p<0.01

With regard to the high ICC values (0.98–1.00) that explain 96–100% of the fluctuation, the correlation of the results between the individual trials was very high in all monitored parameters [45]. The differences found between the individual trials of the measured parameters were not objectively significant, which is confirmed by the results of the effect size. The value of Cohen’s *d* did not exceed 0.2 in any of the cases. The accuracy of measurement can also be expressed by the percentage ratio of the resulting TEM of the measured parameter (Table 3) to the total value of the measured parameter (Table 1 Trial 1). The mean error was 0.38% in males and 0.21% in females for BM, 3.84% in males and 1.63% in females for BF (kg), 3.76% in males and 1.68% in females for BF (%), 0.70% in males and 0.59% in females for FFM (kg), 0.66% in males and 0.87% in females for BMC (kg), 0.78% in males and 0.86% in females for BMD (g/cm^2^). The results of the body weight measurement and all soft tissue parameters in women indicate a significantly lower error based on the comparison of TEM values. The statistical significance in FFM (kg) was p<0.05 and it was p<0.01 in other parameters.

With regard to the low differences in the mean values of the measured parameters of the segmental analysis between the right and left limbs (Table 2), we only present the results of the measurement accuracy for the right limbs and the trunk (Table 4).

The ICC in the segmental analysis of women were similar to the whole-body analysis. They ranged from 0.95 to 0.99 and they explain 90–98% of the ICC fluctuation; the correlation of the results was very high [45]. The soft tissue ICC values in men, RARM an RLEG (except for RLEG BF %) ranged from 0.82 to 0.89 and they explain 67–79% of the fluctuation; the correlation of the results RARM and RLEG in soft tissues (except for RLEG BF %) was high [45]. In other male subjects’ segments and parameters, the ICC values ranged from 0.90 to 0.98 and they explain 81–96% of the fluctuation; the correlation of the results was very high [45]. The results of the effect size did not confirm any practically significant differences between the individual measurements of the parameters. Identically to the whole-body analysis, the value of Cohen’s *d* did not exceed 0.2 in any of the cases. The percentage ratio of the resulting TEMs of the measured parameters (Table 4) to the total values of the measured parameters (Table 2 Trial 1) was in male subjects 5.71–14.49% and in female subjects 4.46–5.41% for BF (kg), 4.11–12.94% in males and 2.17–4.67% in females for BF (%), 2.99–4.77% in males and 2.03–4.00% in females for FFM (kg), 2.27–4.76% in males and 0.00–3.03% in females for BMC (kg), 1.32–2.20% in males and 0.79–2.06% in females for BMD (g/cm^2^).

Similarly to the whole-body analysis, the TEM values in soft tissues show a lower error of measurement in women than in men. The statistical significance of these parameters was p<0.01, with the exception of the BF values of the body, where no statistical significance was determined.

## Discussion

The potential applications of determined TEM values can be demonstrated using results of studies that assess changes in body composition of athletes in various sports throughout the competition season [46–48]. In these studies, researchers assessed the changes in body composition using the DXA method in handball players in preseason and postseason [47], rugby players in preseason, midseason and end-season [48] and softball, basketball and volleyball players, swimmers, and track and field athletes in off-season, preseason and postseason [46]. In many cases, the differences found, even when statistically significant, remain in a range of the TEM (or the upper bound of the confidence interval) or are very close to those values. In such cases, the changes present in the results could be considered negligible.

Based on the results of the whole-body analysis presented in Table 3, the current study assume that the bone parameters (BMC, BMD) are most accurately measured, as they have the lowest TEM values. It corresponds with the fact that DXA system uses different X-ray absorptivity with two pulse levels through the soft tissue and bones [49]. However, the percentage ratio of the resulting TEM to the total value implies that the BM (0.21% in females, 0.38% in males) is determined with the lowest error. The BM value is given by the sum of the individual masses of the individually measured tissues. The correlation between the values measured by the digital scale (InBody 370) and the DXA was high in the individual measurements. The Pearson correlation coefficient values were at the level of *r* = 0.99; this value explains 98% of the fluctuation, which we consider to be a very high correlation of results [45]. Also, the FFM value in female subjects is determined with a lower error than BMC and BMD. In all these parameters, however, the error is below 1%, which can be considered negligible with regard to the error of the method and biological variability, except for BF where the error found is greater (BF kg 1.64% for females and 3.84% for males, BF% 1.64% for females and 3.76% for males). As far as the segmental analysis is concerned, the values of the bone parameters (BMC, BMD) were measured with the lowest error and thus most accurately. A substantial majority of the found measurement errors, up to the determination of the BMC and BMD RLEG RARM in women, were greater than 1%. The BMD errors of determination were significantly lower than the error of the BMC determining (with the exception of 0% BMC RARM for females). Also shows that the errors of BMD and BMC segments determination were higher than whole body estimation errors, in spite of the fact that the laboratory technician is experienced and does not have to position the measured person in any special way, required, for example, in the hip or pelvic measurements.

We did not find any relevant studies in the available sources that would deal with the reliability of measurement in a segmental analysis or that would compare the reliability of measurement between sexes. The studies mostly focus on the other aspects of measurement using the DXA method, as presented in the introduction [18,20,34–37]. Current investigation compared results with the study by Vincente-Rodrígez et al. [50], in which the authors deal with the accuracy of measurement of BMC and BF in young women in two consecutive repeated measurements using the Hologic QDR equipment. The authors state that an error of measurement in BMC is 37.08 g, which is a percentage ratio of 1.78% to the total value. The TEM values determined in current study are lower, whether they are values of individual measurement pairs or the mean TEM value. Even the values of the upper bound of the confidence interval that measured in current investigation do not reach the error stated by the authors (Table 3). An important outcome of the Vincente-Rodrígez et al. study is that authors demonstrated the stability of the error of measurement in BMC as they did not find it increasing when measuring with a one-day interval. The TEM BF % (0.44%) value we determined in the whole-body analysis (Table 3) is within the range of the values presented in the stated study [50]. The authors measured TEM BF % at 0.47% in two repeated consecutive measurements and BF % at 0.43% in one-day interval measurement. The invariability of the error of measurement and the results of the BF value were confirmed. We found two current studies that deal with the issue of the reliability of measurement by the DXA method. Both studies used the Lunar Prodigy device. Schubert et al. [51] studied the error of measurement in soft tissues in 32 young men and women. The study states the value of 0.45% for BF (%) and 0.34 kg for BF (kg). These values are within the range of the values determined in current study, both male and female. However, the error of measurement of 0.72 kg in FFM is higher than the errors determined in the current study. This value is even higher than the value of the top reliability interval limit determined in current investigation. The potential difference may be caused by the different protocol of measurement and hydration that was only monitored 24 hours prior to measurement in our study. Tinsley et al. [52] expressed the error of measurement in percentage in repeated measurement in 17 male athletes and 10 female athletes. The study states an error of 0.5% for BM, < 1% for FFM, 1.2% for BMC and 3% for BF. The values for BM, BMC and BF determined in current study (in female subjects) were lower, the values for BMC were within the range of the error stated by the authors, and they were only higher for BF (in males). The differences found may be caused by the different homogeneity of the studied groups, or by different level of physical fitness.

The reproducibility of the measurement can also be expressed using the correlation of values from repeated measurements. The ICC values that determined current study from the whole-body analysis, ranging from 0.98 to 1.00 (Table 3), were within the boundaries of the values that were presented in other studies. The studies state values from 0.98 to 1.00 independently of the device used [40,51,53], thus confirming the reliability of the method. Similar results were also presented in a study that assessed reliability with an interval of two weeks. The values ranged from 0.96 to 1.00 [39], which also confirms the invariability of the accuracy of measurement.

The values of basic anthropometric parameters for women and men are in accordance with the data in the literature, such as Heyward, Wagner [49]. They confirm a higher amount of muscle mass in men than in women, which is not only the consequence of general sex differences but also of the higher ratio of muscle-based activities in men, which may influence the BMD values [49,52]. Skeletal muscles and bones form a functional unit with the task of ensuring the mobility and stability of the individual. The results of the most recent studies suggest a complex interaction of muscle and bone. A frequently assessed parameter to describe this interaction is the BMC determined by DXA in relation to the FFM. In general, modifications in BMD were statistically relevant in boys and girls. In adults, cross-sectional studies have identified that martial arts are related to higher BMD, which is more evident in men than women [54]. The existence of sex differences on the osteogenic effect attributed to exercise is not completely clear, but biological maturation seems to affect it differently in boys and girls.

Scan analysis and regional segmentations are reliant upon image quality which is a product of subject positioning during the scanning process. As composite mass was assigned to the scanned image on a pixel-by-pixel basis [55], outcomes are influenced by the quantity and distribution of mass viewable in the frontal plane [56]. Presently, no model exists for appendicular segmental analyses of the upper and lower extremities using DXA. Several authors have attempted to differentiate between segments of the extremities [55–58]. However, anatomical inconsistencies exist regarding locations for body segmentation, further confounded by inadequate descriptions for the purpose of reproduction in practical or research contexts.

Because DXA is a valid measurement for quantifying lean, fat, and bone mass and density, DXA methods estimate BSIPs with a greater degree of accuracy than indirect methods for a variety of subject populations [58]. For instance, Rossi et al. [59] found differences in BSIPs between DXA estimates and those of several indirect methods. In particular, differences were the largest for mass moment of inertia estimates, with errors greater than 10% for each indirect method for almost all segments. Especially, the magnitude of these differences was greater in female and male elite swimmers compared with similarly aged adult males who were not competitive athletes.

Positioning and analysis methodologies presented in this paper resulted in very high, nearly perfect reliability when examining hard- and soft- tissue masses across all segments of the upper and lower extremity. While no observable difference in reliability was evident between upper-body and lower-body segments; hard-tissue achieved greater reliability than soft-tissue masses throughout.

### Limitations of the study

There were a few limitations in this study, primarily due to the nature of the DXA scan performed. First, all participants were lying flat instead of standing upright; thus, small shifts in mass locations likely occurred, introducing potential errors in the computation of the DXA parameters (lying flat vs standing). The second main limitation is due to the sample size used. As the participants were selected based on age and physical fitness state from a larger group of students, these results may not be fully representative for the population’s age and physical fitness ranges; however, they still indicate the importance of selecting appropriate trunk segment definitions based on the applications. Evaluated subject’s biological variation including changes in tissue hydration, as well as gastrointestinal tract contents (the microbiome and undigested dietary components) may significantly influent the variation in subject DXA estimates.

## Conclusions

The positioning of the study participants and analysis methodologies implemented in the experiment resulted in very high, nearly perfect reliability when examining hard and soft tissue masses across all segments of the upper and lower extremities. No observable difference in reliability was evident between the upper-body and lower-body segments; hard-tissue masses achieved greater reliability than soft-tissue masses throughout. The percentage ratio of the resulting TEM measured parameter to the total value of the measured parameter showed that the whole-body DXA analysis provides a more accurate value than segmental analysis for both soft and hard tissues. Therefore, this should be respected when using this method in practice. The TEM values of soft tissues show a lower error of measurement in women than in men in the whole-body and segmental analysis. The position of the subject has to be precisely fixated to ensure reproducibility of the DXA analysis results. The knowledge of TEM values is critical when interpreting outcomes of repeated measurements. Only the changes that are greater than TEM, or the upper bound (95%) of the confidence interval of the measurement, can be considered true changes. The reproducibility of the DXA items determination showed no statistical difference between genders for the two measurements representing hard tissue.

## Supporting information

S1 File Source (relevant) data.xlsx. (XLSX)

## Abbreviations

BSIPs: body segment inertial parameters
BM: body mass
BF: body fat
BMC: bone mineral content
BMD: bone mineral density
DXA: dual-energy X-ray absorptiometry
FFM: fat free mass
M: mean
SD: standard deviation
TEM: typical error of measurement
ICC: intraclass correlation
95% CI: confidence interval typical error of measurement
LARM: left upper limb
RARM: right upper limb
LLEG: left lower limb
RLEG: right lower limb
